# Acute epiglottitis in an older patient: a case report and review of the literature

**DOI:** 10.1186/s13256-025-05363-3

**Published:** 2025-10-27

**Authors:** Eduardo J. Correa, Antonio Sanmartín Caballero, Carlos Almagro Ordóñez, Adrian Carballada Formichelli, Diego M. Conti

**Affiliations:** 1 Departamento de Otorrinolaringología, Quironsalud Marbella y Hospital Campo de Gibraltar, 29603 Marbella, Spain; 2 Otolaryngology Department, Hospital Comarcal de la Línea de La Concepción, Cádiz, Spain; 3 Otolaryngology Department, Hospital Universitario de Puerto Real, Cádiz, Spain; 4 Escuela de Doctorado UAM, Centro de Estudios de Posgrado, Universidad Autónoma de Madrid, Ciudad Universitaria de Cantoblanco, Calle Francisco Tomás y Valiente, No. 2, 28049 Madrid, Spain; 5 Allergy and Clinical Immunology Research Unit, Department of Microbiology and Immunology, KU Leuven, Louvain, Belgium

**Keywords:** Acute epiglottitis, Adult epiglottitis, Supraglottitis, Airway intervention

## Abstract

**Background:**

Acute epiglottitis is a rapidly progressive infection of the supraglottic structures that can potentially be life-threatening. Although its incidence has decreased in pediatric populations owing to widespread vaccination, it remains a diagnostic and therapeutic challenge in adults, where clinical presentations may be subtle, and airway compromise can occur with little warning. The case of a 64-year-old white European female patient is presented.

**Case presentation:**

The case of a 64-year-old white European female patient is presented. Initially, she exhibited symptoms consistent with a foreign body sensation. However, these symptoms rapidly progressed to the classic signs of acute epiglottitis. The patient was managed conservatively, and no invasive treatment was required. In addition, a comprehensive literature review is hereby presented, addressing the salient aspects of this challenging disease. These aspects include but are not limited to epidemiology, etiologic agents, clinical presentation, complementary diagnostics, and advanced management.

**Conclusion:**

Acute epiglottitis is a medical emergency with a rapid progression and severe consequences, thus requiring a high index of clinical suspicion in patients presenting with nonspecific symptoms. A multidisciplinary approach and immediate therapeutic measures are imperative to ensure the safety of the airway through basic and invasive procedures, in addition to strict postprocedural follow-up. This approach is fundamental to optimize health outcomes among these patients.

## Introduction

Acute epiglottitis (AE) represents a life-threatening medical emergency, characterized by a significant risk of upper airway obstruction [1]. Consequently, a multidisciplinary approach, immediate therapeutic measures and a strict close follow-up are required [1].

In this paper, we present the case of a 66-year-old white European female who exhibited clinical manifestations similar to a foreign body, with rapid progression to AE. Furthermore, we review literature on this population and discuss the management of this disease.

## Case report

A 64-year-old white European female patient with a history of hypertension and a previous smoking habit that ceased 30 years ago presented to the emergency room with a suspicion of a foreign body after consuming fish. No other pertinent medical history was provided. The patient reported a sore throat, dry cough, sensation of foreign body in the throat, and mild difficulty swallowing. Upon examination, erythema was observed on the left pharyngeal posterior pillar, and flexible laryngoscopy revealed erythema on the homolateral arytenoid. A combination of paracetamol and dietary measures was prescribed.

The patient returned the following day, reporting an exacerbation of dysphagia and sialorrhea, accompanied by a sensation of neck swelling. Palpation of the neck revealed no abnormalities. The oropharyngeal examination revealed a persistence of erythema, as observed the previous day. However, flexible laryngoscopy revealed an erythematous, tense, and swollen epiglottis. The Cormack–Lehane score [1] was four, indicating impairment in visualizing the vocal cords. It was evident that the patient was suffering from AE; hence, the decision was taken to admit the patient ward for therapeutic treatment.

The absence of fever, heart rate of 72/min, respiratory rate of 17/min, and oxygen saturation of 96% indicated no systemic involvement.

The blood test revealed a white blood cell (WBC) count of 12.36 μL, with a predominance of neutrophils, a glucose level of 116 mg/dL, and a C-reactive protein level of 82.2 mg/L (within the normal range of 0–5).

The patient was prescribed intravenous ceftriaxone, hydrocortisone, oral methylprednisolone, noninvasive mask ventilation with oxygen, and nebulised budesonide, under strict monitoring.

Initially, the patient presented signs of mild improvement, with no evidence of airway obstruction or respiratory impairment. Laryngoscopy revealed a Comarck 3, which we interpreted as an indication of improvement. Following the third day, the patient exhibited significant clinical improvement, and we proceeded with the regular laryngoscopy.

**Fig. 1:**
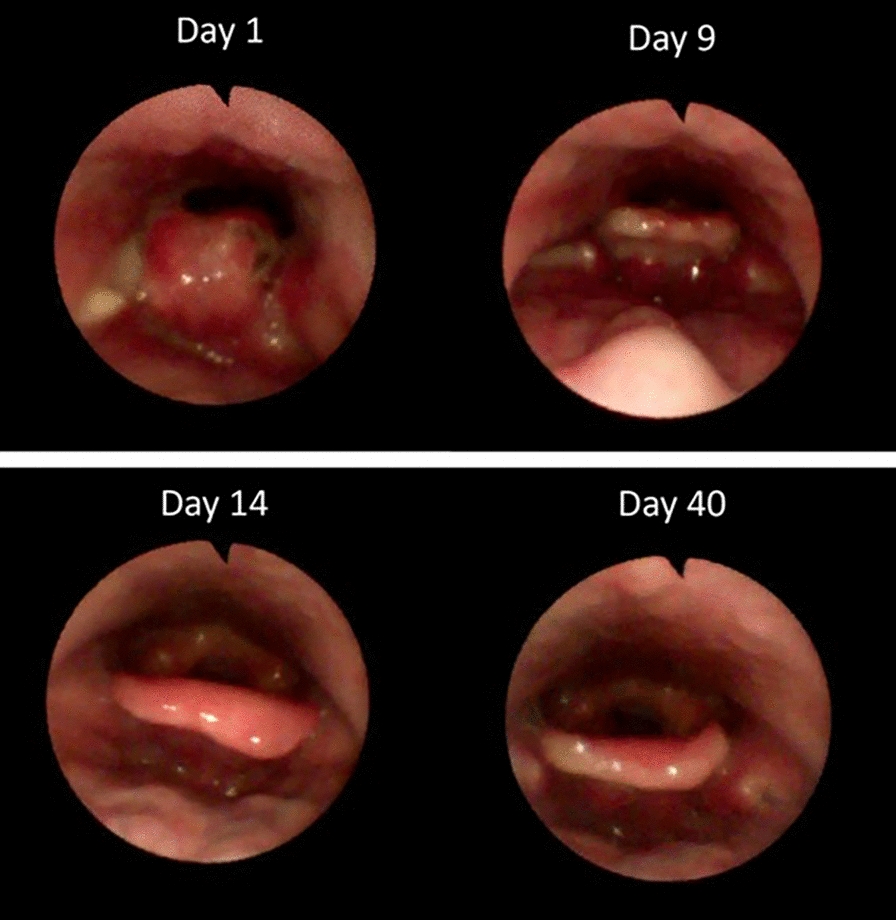
Laryngoscopic evolution from day 1 to day 40

On day 9, the patient was asymptomatic, with vital signs within normal parameters. Laboratory tests revealed a WBC count of 6.47 µL, a C-reactive protein level of 52 mg/L, and a procalcitonin level of 0.04 ng/mL, indicating the absence of bacterial infection. The patient was discharged with a prescription for oral antibiotics and a reduced dosage of oral steroids, with instructions to attend an outpatient clinic for follow-up and periodic laryngoscopic assessment. Figure 1 illustrates the evolution of the laryngoscopic examination.

## Discussion

AE represents a medical emergency characterized by the rapid onset of inflammation of the epiglottis and supraglottic mucosa. This can result in airway obstruction and ventilatory impairment, with a mortality rate of 1–20% [1, 2]. The condition may present with symptoms including sore throat, dysphagia, drooling, a muffled voice, and dyspnea [3].

As with any infection, it can manifest with systemic repercussions, including tachycardia, fever, and poor glucose control.

It is regarded as a bacterial infection, predominantly caused by *Haemophilus influenzae*, *Streptococcus pneumoniae*, and *Staphylococcus aureus* [1–3]. This condition presents primarily in childhood with acute respiratory distress and fever, with a declining incidence due to the implementation of *Haemophilus influenzae* vaccination programmes. Adults frequently present with dysphagia symptoms, with an incidence of 1–4 per 100,000 [4] and a male-to-female ratio of 2.02:1 [1–4]. The age of presentation in adults varies considerably, from 19 to 96 years old [1–4].

A literature review revealed an increased incidence of AE in 2019 [1–4] in conjunction with the presence of comorbidities, including hypertension, diabetes, and obstructive sleep apnea [1–4].

AE diagnosis is based on clinical evaluation. Although a culture sample can provide a precise microbiological diagnosis, the supraglottis is a reactive structure, and manipulation of the zone can lead to a rapid onset of airway obstruction. Consequently, empiric treatment is recommended. Whilst complementary imaging procedures are described (lateral neck radiograph or computed tomography (CT) scan) [5], it is our position that, given the progressive nature of the airway obstruction, immediate therapeutic measures should be implemented if suspected. Flexible laryngoscopy, if available, can be used to confirm the diagnosis with certainty. This concept is corroborated by other authors [4, 5].

Therapeutically, securing the airway is of paramount importance as this inflammatory condition has the potential to cause significant airway obstruction with minimal warning. In our case, we chose to administer oxygen via a noninvasive ventilation mask, as her general condition showed normal arterial blood saturation levels, no tachycardia, and no tachypnea. Securing ventilation may necessitate aggressive procedures, such as orotracheal intubation or tracheostomy. A number of authors identified a number of factors that may indicate the necessity for more aggressive intervention. These include subjective dyspnea and/or objective respiratory effort, respiratory distress, stridor, elevated C-reactive protein, older age, body mass index greater than 25 kg/m^2^, and a history of diabetes mellitus [4, 5].

Combined intravenous corticosteroids and antibiotics are mandatory. In the various published studies, the selection of methylprednisolone, hydrocortisone, and the antibiotic regimen differed between ceftriaxone, ampicillin–sulbactam, clindamycin, cefuroxime, and metronidazole [4, 5]. Initial treatment regimen consisted of intravenous ceftriaxone and hydrocortisone, with oral methylprednisolone as adjunct, as determined through an interdisciplinary process involving internal medicine and infectious disease specialists at our institution. Additionally, nebulized hydrocortisone was administered for the initial 2 days of admission. On the third day, a clinical improvement was observed, prompting a switch to oral dexamethasone.

An interdisciplinary approach is essential in the management of AE, necessitating the involvement of at least otolaryngology, internal medicine, infectious diseases, and nursery.

## Conclusion

AE represents a medical emergency with rapid onset and severe consequences. Multidisciplinary approach and immediate therapeutic measures are required, including oxygen ventilation, steroids, and antibiotics. Securing the airway is of the utmost importance; therefore, healthcare professionals must be prepared to perform invasive procedures such as orotracheal intubation and tracheostomy.

## Abbreviations

AE: Acute epiglottitis
WBC: White blood cell

## Declarations

### Availability of data and materials

The datasets used and/or analyzed during the current study are available from the corresponding author on reasonable request.
